# Temporal diabetes-induced biochemical changes in distinctive layers of mouse retina

**DOI:** 10.1038/s41598-018-19425-8

**Published:** 2018-01-18

**Authors:** Ebrahim Aboualizadeh, Christine M. Sorenson, Alex J. Schofield, Miriam Unger, Nader Sheibani, Carol J. Hirschmugl

**Affiliations:** 1 Department of Physics, University of Wisconsin-Milwaukee, Milwaukee, Wisconsin USA; 2 Department of Pediatrics, University of Wisconsin School of Medicine and Public Health, Madison, Wisconsin USA; 3 McPherson Eye Research Institute, University of Wisconsin School of Medicine and Public Health, Madison, Wisconsin USA; 4 Physical Electronics GmbH, Ismaning, Germany; 5 Departments of Ophthalmology and Visual Sciences, Biomedical Engineering, and Cell and Regenerative Biology, University of Wisconsin School of Medicine and Public Health, Madison, Wisconsin USA; 6 Present Address: Center for Visual Science, University of Rochester, Rochester NewYork, USA

## Abstract

To discover the mechanisms underlying the progression of diabetic retinopathy (DR), a more comprehensive understanding of the biomolecular processes in individual retinal cells subjected to hyperglycemia is required. Despite extensive studies, the changes in the biochemistry of retinal layers during the development of DR are not well known. In this study, we aimed to determine a more detailed understanding of the natural history of DR in Akita/+ (type 1 diabetes model) male mice with different duration of diabetes. Employing label-free spatially resolved Fourier transform infrared (FT-IR) chemical imaging engaged with multivariate analysis enabled us to identify temporal-dependent reproducible biomarkers of the individual retinal layers from mice with 6 weeks,12 weeks, 6 months, and 10 months of age. We report, for the first time, the nature of the biochemical alterations over time in the biochemistry of distinctive retinal layers namely photoreceptor retinal layer (PRL), inner nuclear layer (INL), and plexiform layers (OPL, IPL). Moreover, we present the molecular factors associated with the changes in the protein structure and cellular lipids of retinal layers induced by different duration of diabetes. Our paradigm provides a new conceptual framework for a better understanding of the temporal cellular changes underlying the progression of DR.

## Introduction

Diabetic retinopathy (DR) is a progressive retinal neurovascular dysfunction that can ultimately lead to blindness^1^. The various cellular components of the retina, especially the vascular cells, are susceptible to the hyperglycemic environment that triggers unique biochemical and cellular alterations. These cellular changes occur through a number of pathways including, elevated oxidative stress^2^, protein kinase C (PKC) activation^3,4^, and advanced glycation end (AGE) product formation^5^. Oxidative stress is considered as a unifying mechanism that links the existing pathophysiological pathways^6^. Membrane thickening^7^, microvascular cell loss^8^, and capillary closures^9^ are additional structural alterations that are impacted by reactive oxygen species (ROS). Information on dysfunction of neuronal cells of the retina, especially cells with fragmented DNA^10,11^ have also been gaining interest as a primary target of early diabetes changes. Understanding the underlying mechanism of oxidative stress-mediated damage at specific cellular levels, offers potential therapeutic targets that will assist in developing early diagnosis and effective treatment for DR. Despite wide-ranging studies, little is known about the changes in the biochemistry of the individual retinal cell layers during diabetes and their contribution to the pathogenesis of DR.

Diabetic rodents provide a focused study of the impact of hyperglycemia on the pathophysiological machinery involved, and recapitulate human hyperglycemia and several factors that are frequently found in DR. Numerous *in vivo* imaging techniques including fundus photography, optical coherence tomography, and confocal scanning light ophthalmoscopy (cSLO), have been employed to determine the anatomical and morphological information in the neurodegenerative mouse retina^12–14^. There is abundant effort for developing retinal imaging modalities that allows a better assessment of the disease at the earliest stages. Today, technical advances in adaptive optics^15^ facilitated the visualization of the individual neurons in the retina by correcting higher order aberrations, which allows detection of clinically relevant details at the cellular level. Much efforts have also gone towards recording the functionality of the retinal cells such as delivering visual stimuli and recording physiology of ganglion cells by using genetically encoded calcium indicators (G-CaMP)^16^, and fluorescence imaging to label proteins within cells that are transgenically expressed in the living mouse eye^17^. To our knowledge, none of these *in vivo* imaging techniques have been successful in determining the strict biochemical processes in the retinal tissue. Lateral and axial resolutions, depth of penetration in the eye, contrast from imaging modalities, optical sectioning, and the incapability of early intervention, are some restraints that hindered an in-depth understanding of the pathogenesis of DR in microscopic realm^15^. Overcoming these technical limitations would facilitate a better understanding of the natural history of DR.

Histological staining such as Hematoxylin and Eosin has been adapted for analyzing paraffin-embedded and formalin-fixed tissue sections *ex vivo*. However, fixation and staining can distort the architecture and the metabolic state of the tissue, in some cases leading to a biased assessment of the native biochemistry of the tissue. Fourier transform infrared (FT-IR) chemical imaging is a label-free and non-destructive technique that permits detecting the inherent vibrational properties of the constituents of cells and tissues^18–22^. Spatially resolved chemical images from this technique allow the simultaneous visualization of multiple retinal layers and characterization of localized biochemical changes in the tissue without applying exogenous stains or dyes. The study of the bio-molecular alterations in the biochemistry of retinal layers with different duration of diabetes would be greatly facilitated by vibrational spectroscopic techniques without any need for isolating the cell compartments. FT-IR chemical imaging has been used to investigate various tissue types including adipose^23^, prostate^24^, breast^25,26^, kidney^27^, brain^28,29^, pancreas^30^, pleura^31^, and colon^32^.

**Figure 1:**
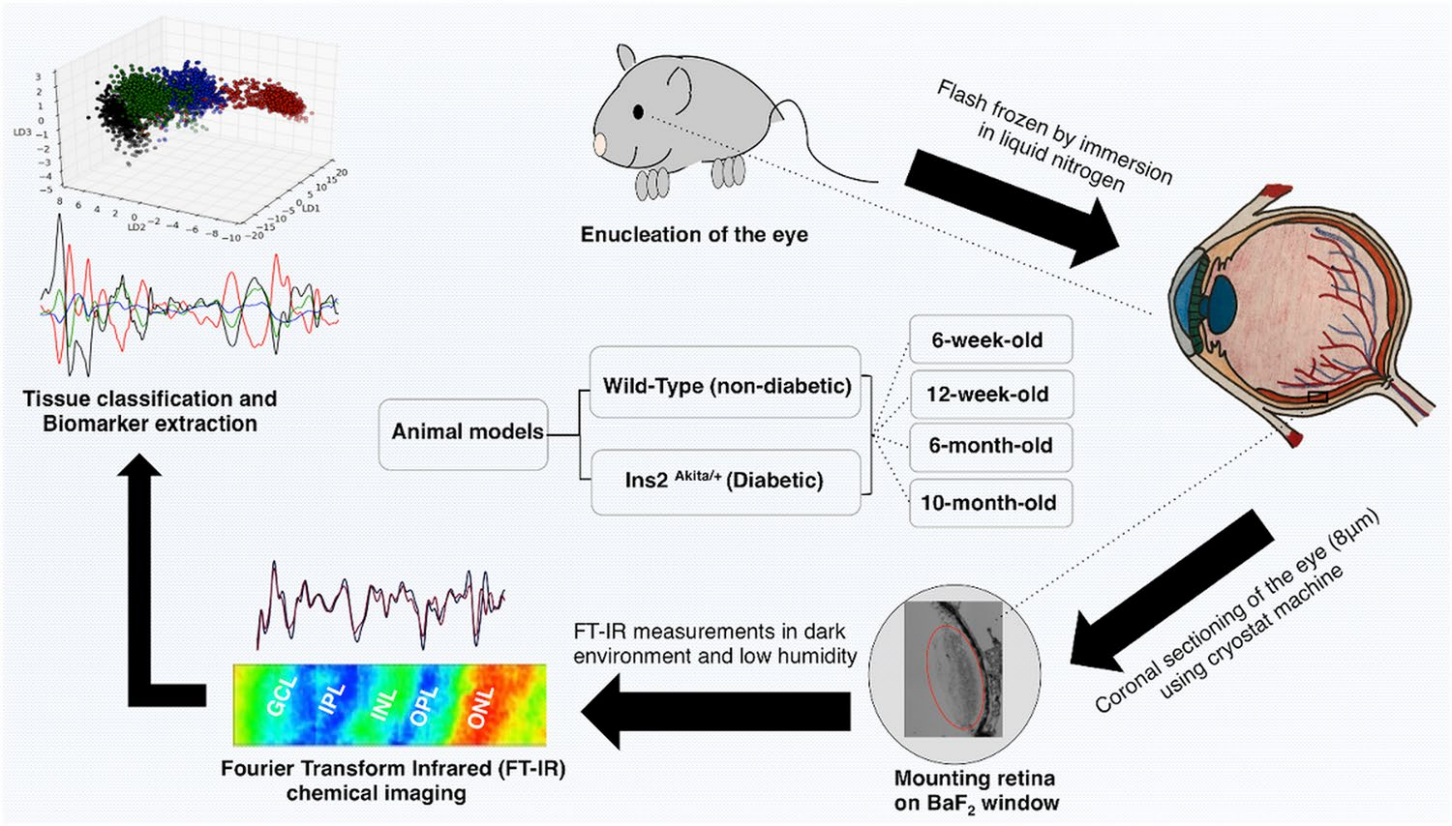
Schematic illustration of the experimental design.

Recently, we reported the significance of photoreceptors in the oxidative stress damage to the retina at the onset of diabetes and the biomarkers for each retinal layer were achieved^33^. In this article, for the first time, we demonstrated that FT-IR chemical imaging is a promising approach to quantifying temporal-dependent biomolecular changes induced by diabetes in distinctive layers of mouse retina. Several morphological and cellular retinal lesions in the Akita/+ mice with different duration of diabetes have been reported^34,35^. We aimed to characterize the biochemical changes at 6 and 12 weeks, 6 and 10 months of age based on the significance of the reported timepoints in previous studies (the experimental design is shown in Fig. 1). Intra-layer comparison between photoreceptor layer (PRL), outer plexiform layer (OPL), inner nuclear layer (INL), and inner plexiform layer (IPL), at different duration of diabetes was performed and the associated biomarkers were determined. Here, we also report the biochemical changes in the individual layers of retina from Akita/+ mice, as a function of the progression of diabetes. These results provide novel insight into the cellular specific retinal changes during the development and progression of DR.

## Results

### Classification of retinal tissues from wild type and Akita/+ mice

**Figure 2:**
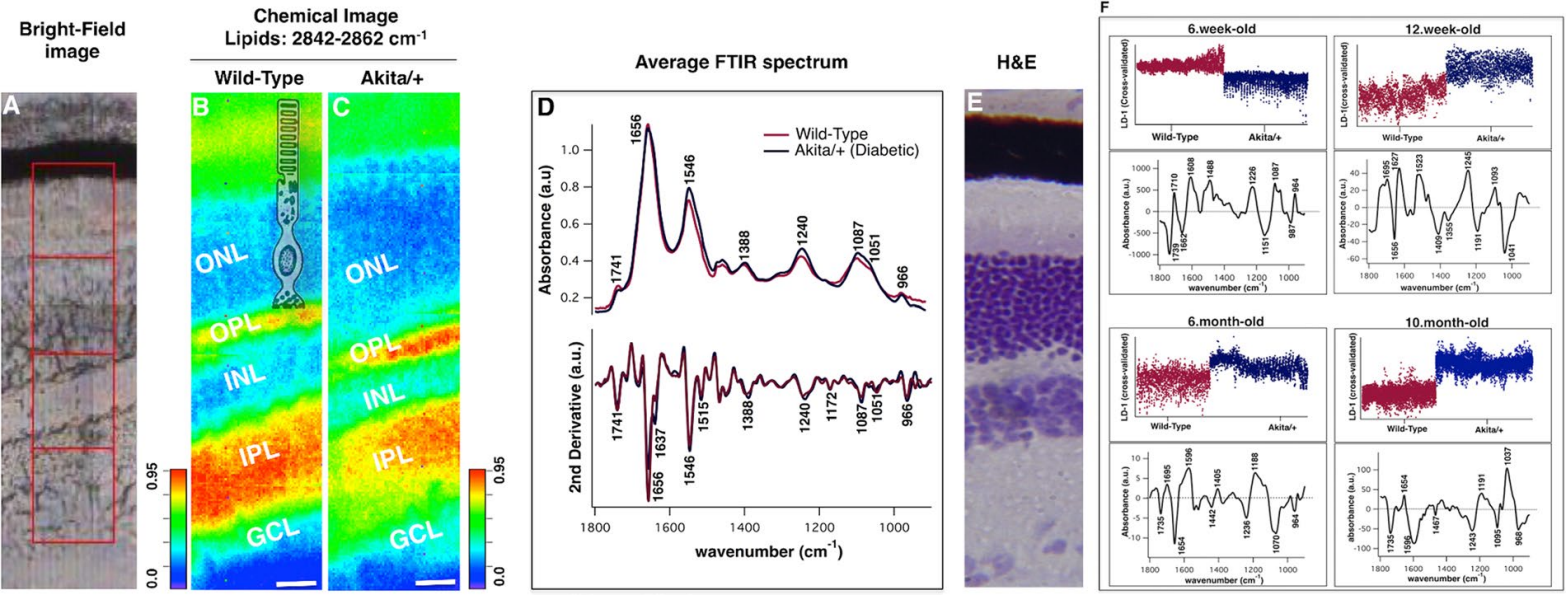
Bright-field image (**A**), and FT-IR chemical images of coronal retinal tissue from 12-week-old wild type (**B**) and Akita/+ (**C**) mice highlighting the microstructure of retina. Data is representative. FT-IR chemical image covers the area of ≈70 × 270 μm^2^ (1 mosaic, 4 tiles (each tile = 64 × 64 pixels)), integrated for the lipid band at 2842–2862 cm^−1^ to show lipid distribution. Panel C has been overlaid with the sketch of the photoreceptor system including rods, mitochondria, and the nucleus. The scale bar is 20 μm and colors indicate component gradients from low (blue) to high (red). (**D**) Averaged and normalized second derivative spectra of retinal tissues from wild type and Akita/ + mice in the spectral range 1800–900 cm^−1^ are shown. An average of ≈16,000 individual pixel spectra from all pixels per tissue section and 5 retinal sections per genotype, is demonstrated. (**E**) H&E stained tissue section to highlight the heterogeneity of the retinal structure (Reprinted from ref.^18^, Copyright (2012), with permission from Elsevier). Data are representative. (**F**) Comparison of the tissues from WT and Akita/+ animals at 6, 12 weeks and 6, 10 months of age, generated from PCA–LDA. One-dimensional cross-validated scores plot and the corresponding spectral loading plot highlighting major differentiating frequencies are demonstrated at different duration of diabetes. Each dot in the score plot is representative of a pixel (spectrum) within each tissue section. Data is representative of one section per tissue. The 6.week-old Panel in F has been reproduced from ref.^33^ with permission from the Royal Society of Chemistry. ONL, Outer nuclear layer; OPL, Outer plexiform layer; INL, Inner nuclear layer; IPL, Inner plexiform layer; GCL, Ganglion cell layer.

Bright-field and FT-IR chemical images of the retinal tissue from 12-week-old wild type (WT) and Akita/+ mice, oriented vertically from the photoreceptors to the ganglion cell layer, are shown (Fig. 2A–C). Representative data from one coronal section of retinal tissue demonstrates the efficacy of FT-IR chemical imaging in resolving retinal layers. As shown (Fig. 2B and C), a mosaic image covers the area of ≈70 × 280 μm^2^ (1 mosaic, 1 × 4 tiles (each tile = 64 × 64 pixels), pixel resolution: 1.1 μm), and integrated over the lipid band at 2850 cm^−1^ to highlight the individual retinal layers and facilitate understanding of morphology. Lipid distribution is shown as a false-color image (Red – blue: high – low concentration) i.e. regions of greatest lipid content (plexiform layers) are red and regions of lowest lipid content containing nuclear bodies (ONL and INL) are blue across the retinal tissue. A chemical image (Fig. 2B) is overlaid with the sketch of photoreceptor system including rod cells, mitochondria, and nucleus. Spectra representative of the distinct biochemistry of retinal layers are unequivocally discussed elsewhere^18^. Averages from nearly 16,000 individual pixel spectra from the entire tissues (WT and Akita/+) are overlaid (Fig. 2D). To better resolve the peak positions and subtle differences in functional groups between the tissues, we include a second derivative analysis (Savitzky-Golay algorithm, 9 smoothing points). Figure 2D reveals apparent differences in the intensity of FT-IR signals between 1000 and 1300 cm^−1^, predominated by ν (C-O, C-C, C-O-C) and ν_sym, asym_ (PO^2−^), and between 1500 and 1600 cm^−1^, mainly associated with amide II groups of proteins. The biomolecular assignments of the bands for the retinal tissue were delineated in our previous study^33^. Figure 2E shows a coronal section of the retinal tissue stained with hematoxylin and eosin (H & E) to highlight the microstructural organization of the tissue (Reprinted from ref.^18^, Copyright (2012), with permission from Elsevier).

Retinal tissues prepared from WT and Akita/+ mice at 6 and 12 weeks, and 6 and 10 months of age, were compared (Fig. 2F) using principal component analysis followed by linear discriminant analysis (PCA-LDA) as described in the methods below. PCA-LDA score plots (Fig. 2F) show a very good degree of separation between the two groups of interest, namely WT and Akita/+ at 6 weeks, 12 weeks, 6 months, and 10 months of age. The PCA-LDA cluster vector plots (Fig. 2E) revealed the major discriminating absorption signatures as a function of wavenumber that are responsible for the classification between the tissues at each stage of diabetes. The scores for WT group at 12 weeks, 6 months, and 10 months of age were lower than the scores for the Akita/+ group at the related ages. However, the scores for WT group at 6 weeks of age were higher than the scores for the Akita/+ group at the associated ages. The scores in the scatterplot (for each class) are based on the algorithm training. The list of differentiating bands and their biomolecular assignments are summarized in Table 1.

**Table 1:** Discriminatory frequencies (cm^−1^) from PCA-LDA and associated biomolecular assignments when the entire retinal tissue (including all retinal layers) from WT and Akita/+ mice was compared at 6 weeks, 12 weeks, 6 months, and 10 months of age. The biomolecular assignments acquired from references^56–59^.

6-week-old	12-week-old	6-month-old	10-month-old	Molecular Assignment
1739		1735	1735	Lipids: C=O ester in phospholipids
1710				Nucleic acids: Base pair carbonyl (C=O), nucleic acids, DNA, RNA, oxidation of cellular lipids, fatty acids
	1695	1695		Proteins: Amide I- Antiparallel *β*-sheet
1662				Proteins: Amide I- turns 3_10_ helical structure of proteins (mainly C-O stretching; contribution from C-N stretching)
	1656	1654	1654	Proteins: Amide I- α-helical structure
	1627			Proteins: Amide I- β-sheet structure
1608				Proteins: Amide I- aggregated strands
		1596	1596	Proteins: Amide II- mainly N-H bending
	1523			Proteins: Amide II- β-sheet structure
		1442	1467	Lipids: CH_2_ bending
	1409	1405		Lipids: C-H deformation
	1355			Stretching C-O, deformation C-H, deformation N-H
1226	1245	1236	1243	Nucleic acids: Phosphates: ν_asym_ PO^2**−**^
	1191		1191	Phosphate (P=O) band; collagen
		1188		Deoxyribose
1151				C-O, C-C stretching, C-O-H, C-O-C deformation of carbohydrates
1087	1093		1095	Nucleic acids: phosphates: ν_sym_ PO^2**−**^
		1070		C-O stretching, deoxyribose/ribose, DNA, RNA
	1041		1037	C–O stretching, ribose
987				Nucleic acids and proteins, protein phosphorylation, OCH_3_ (polysaccharides-cellulose)
964		964	968	Nucleic acids, symmetric PO^4−^ stretching (DNA) and deoxyribose; phosphate skeletal motions

### Intra-retinal layer classification at different duration of diabetes

**Figure 3:**
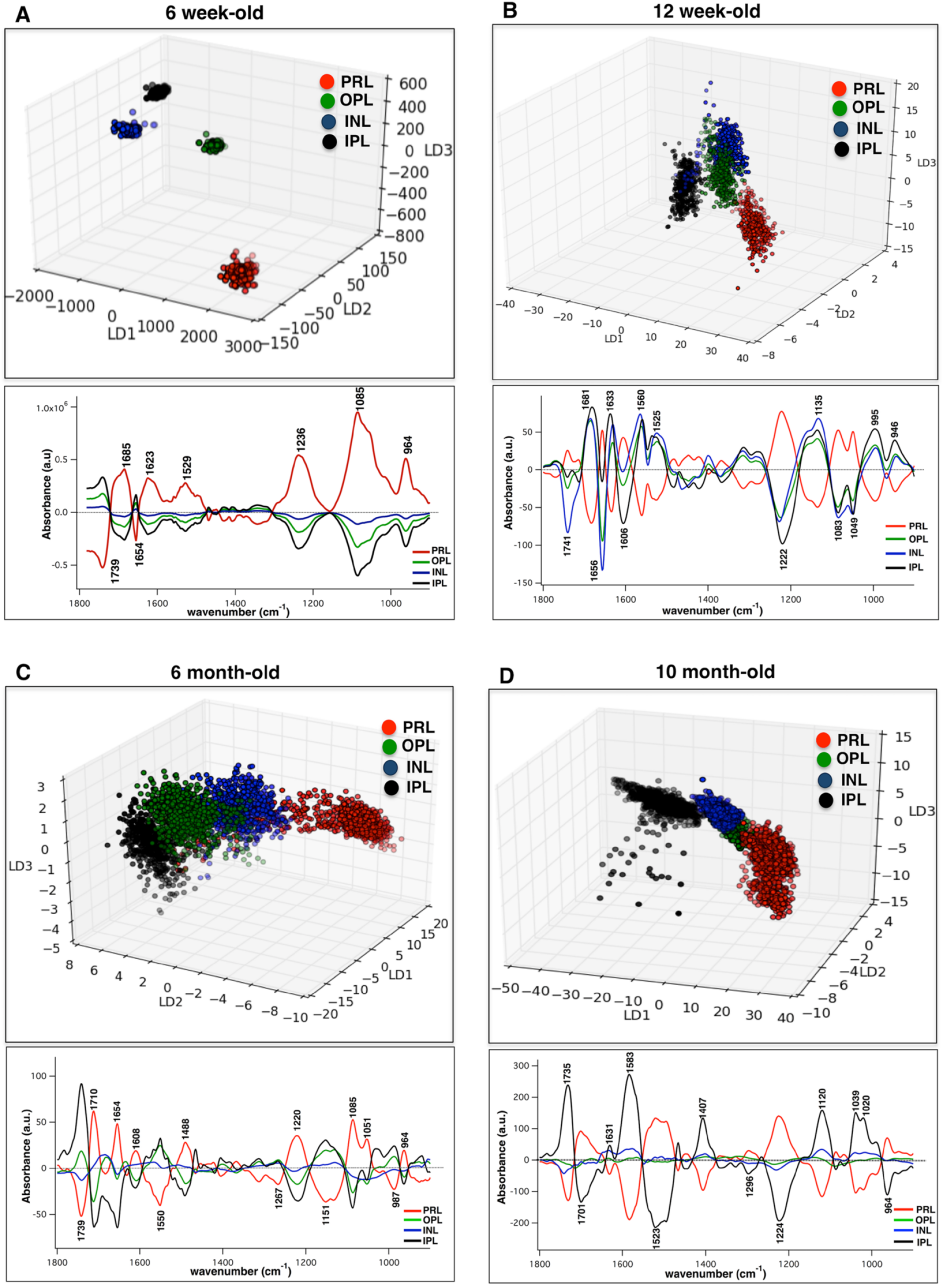
Intra-retinal layer classification at different duration of diabetes. Comparison of distinctive diabetic retinal layers, namely PRL, OPL, INL, and IPL at 6 weeks (**A**) This Panel has been reproduced from ref.^33^ with permission from the Royal Society of Chemistry), 12 weeks (**B**), 6 months (**C**), and 10 months (**D**) of diabetes derived from PCA-LDA. Three-dimensional rotated scores plot and cluster vector plot highlighting discriminatory frequencies (cm^−1^) are demonstrated for each case study. Nearly 1500 spectra from each layer of the retina, per tissue section/age were generated and compared. Each dot in the scores plot is representative of a pixel (spectrum) within attributed layer of the retina from Akita/+ mice. Data is representative. The analysis was repeated for at least 5 tissue sections per animal. N = 6; N is the number of animals. PRL, Photoreceptor retinal layer; OPL, Outer plexiform layer; INL, Inner nuclear layer; IPL, Inner plexiform layer.

To achieve a better understanding of the diabetes-induced impacts on distinctive retinal layers, a comparison was made between the diabetic PRL, OPL, INL and IPL with different duration of diabetes. Nearly 1500 IR spectra were derived from characteristic layers of diabetic tissues and compared using PCA-LDA. Figure 3 shows three-dimensional rotated score plots comparing diabetic retinal layers at 6-weeks (3 A), 12-weeks (3B), 6-months (3 C), and 10-months (3D) of age. The corresponding loading plots that exhibit the spectral biomarkers of intra-retinal layer classifications are shown. Striking segregation between the diabetic retinal layers was achieved; note that the best classification between the layers was obtained at 6 weeks of age (Fig. 3A). Table 2 lists the frequencies of the major signatures with their tentative bio-molecular assignments associated with intra-retinal layer classification. The intra-layer classification between non-diabetic retinal layers was not significantly different (data not shown).

**Table 2:** Discriminatory frequencies (cm^−1^) from PCA-LDA and associated biomolecular assignments from the intra-retinal layer comparison (between PRL, OPL, INL, and IPL from Akita/+ mice) at 6 weeks, 12 weeks, 6 months, and 10 months of age. The biomolecular assignments acquired from references^56–59^ PRL, Photoreceptor retinal layer; OPL, Outer plexiform layer; INL, Inner nuclear layer; IPL, Inner plexiform layer.

6-week-old	12-week-old	6-month-old	10-month-old	Molecular Assignment
1739	1741	1739	1735	Lipids: C=O ester in phospholipids
		1710		Base pair carbonyl (C=O), nucleic acids, DNA, RNA, oxidation of cellular lipids, fatty acids
			1701	Lipids: C=O ester
1685	1681			Proteins: Amide I- turns or antiparallel β-sheet structure (mainly C-O stretching)
1654	1656	1654		Proteins: Amide I- α-helical structure
	1633		1631	Proteins: Amide I: β-sheet structure
1623				Proteins: Amide I- aggregated strand structures (mainly C-O stretching)
	1606	1608		Proteins: Amide I-aggregated strands
			1583	Proteins: Amide II- mainly N=H bending, ring C-C stretch of phenyl
	1560	1550		Proteins: Amide II- mainly N-H bending
1529	1525		1523	Proteins: Amide II- mainly C=N, C=C stretching
			1407	Lipids: C-H deformation
			1296	Proteins: Amide III, mainly N-H bending
		1267		Proteins: Amide III, mainly N-H bending
1236	1222	1220	1224	Nucleic acids: phosphates: ν_asym_ PO^2**−**^
		1151		C-O, C-C stretching, C-O-H, C-O-C deformation of carbohydrates/ glycogen
	1135			C-O, C-C stretching, RNA ribose
			1120	C-O stretching, RNA ribose
1085	1083	1085		Nucleic acids: phosphates: ν_sym_ PO^2**−**^
	1049	1051		C–O stretching, deoxyribose/ribose DNA, RNA
			1039	C–O stretching, ribose
			1020	C-O, C-C stretching, DNA, glycogen,
	995			C-O ribose, C-C, RNA
		987		Nucleic acids and proteins, protein phosphorylation, OCH_3_ (polysaccharides-cellulose)
964	964	964	964	Nucleic acids, symmetric PO^4−^ stretching, (DNA) and deoxyribose; phosphate skeletal motions

### The biochemical changes over time for each individual layer of retina

**Figure 4:**
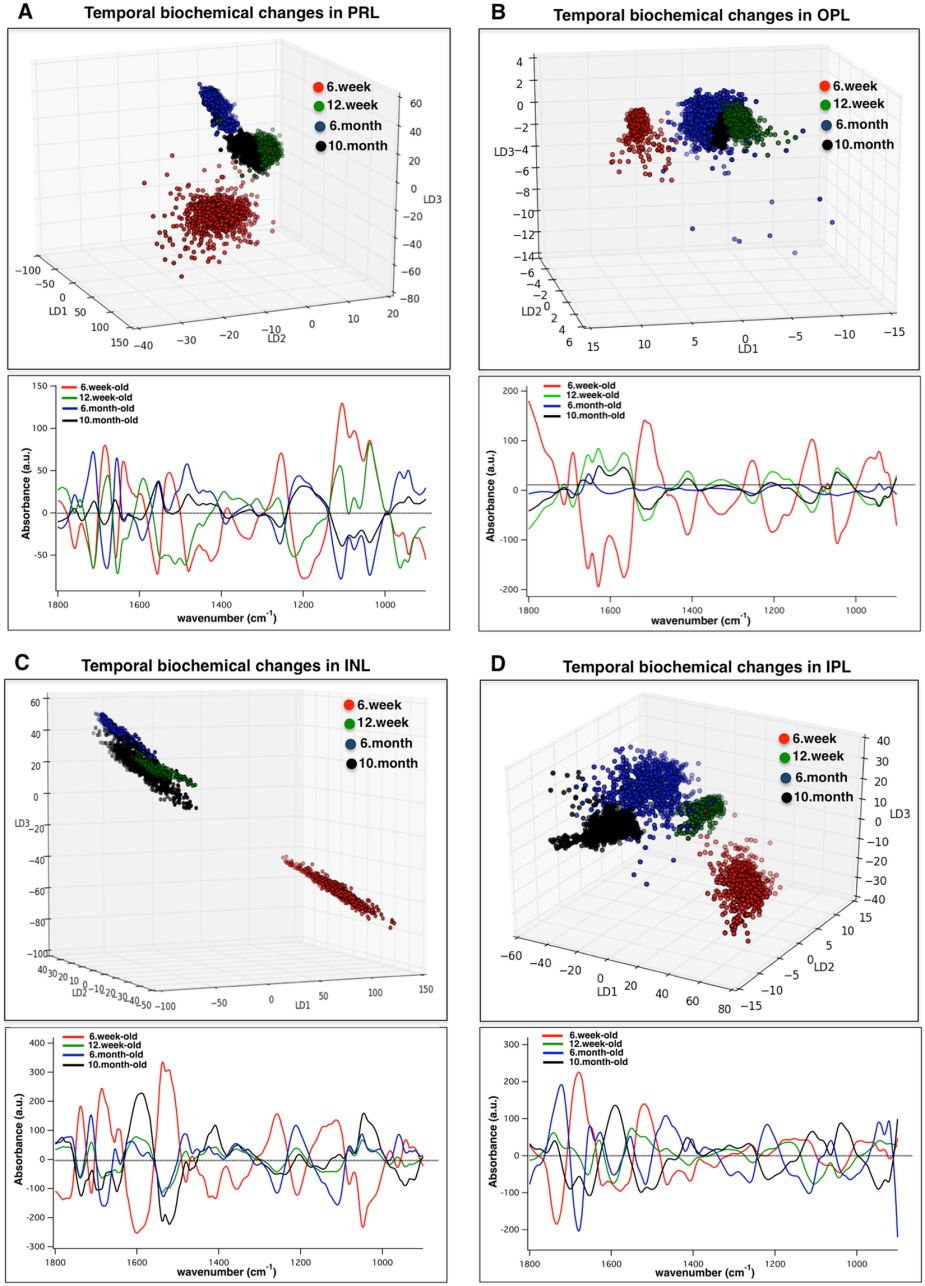
Temporal-dependent diabetes-mediated biochemical alterations for the PRL, OPL, INL, and IPL, as a function of progression of diabetes. Three-dimensional rotated scores plot and cluster vector plot from PCA-LDA are shown. Clustering between spectra from the PRL (**A**), OPL (**B**), INL (**C**), and IPL (**D**), as a function of age. Nearly 1500 spectra were used for the comparison per retinal layer/ per age. Each dot in the scores plot is representative of a pixel (spectrum) within attributed layer of the retina from Akita/+ mice. Data is representative. The analysis was repeated for at least 5 sections per animals. N = 6; N is the number of animals. PRL, Photoreceptor retinal layer; OPL, Outer plexiform layer; INL, Inner nuclear layer; IPL, Inner plexiform layer.

To ascertain the temporal diabetes-mediated changes in the biochemistry of the PRL, OPL, INL, and IPL, we performed PCA-LDA on the 1500 layer-specific spectra (from each age group) to examine how well the spectral signatures can be discriminated as a function of the progression of diabetes. Temporal-dependent three-dimensional rotated score plots and associated loading plots from the comparison of the diabetic PRL (Fig. 4A), OPL (Fig. 4B), INL (Fig. 4C), and IPL (Fig. 4D) are displayed. As demonstrated in Fig. 4, spectra from short-term diabetes (6-week-old) present a clear segregation from the other stages of diabetes, for all retinal layers studied here. Table 3 lists discriminatory frequencies associated with the classifications that describe the temporal-dependent biomarkers of diabetes for each retinal layer.

**Table 3:** Discriminatory frequencies (cm^−1^) from PCA-LDA and associated biomolecular assignments from the temporal-dependent comparisons for each retinal layer (PRL, OPL, INL, IPL), as a function of progression of diabetes (6 weeks, 12 weeks, 6 months, and 10 months). The biomolecular assignments acquired from referencesnd associated biomolecular assignments^56–59^ PRL, Photoreceptor retinal layer; OPL, Outer plexiform layer; INL, Inner nuclear layer; IPL, Inner plexiform layer.

PRL	OPL	INL	IPL	Molecular Assignment
1758				Lipids: C=C stretching
1741		1739	1741	Lipids: C=O ester in phospholipids
1712		1710		Base pair carbonyl (C=O), nucleic acids, DNA, RNA, oxidation of cellular lipids, fatty acids
	1693	1691		Proteins: Amide I- Antiparallel *β*-sheet
1681		1683		Proteins: Amide I- turns or antiparallel β-sheet structure (mainly C-O stretching)
1677			1679	Proteins: Amide I- turns structure (mainly C-O stretching)
1656	1654	1654	1654	Proteins: Amide I- α-helical structure
1637	1629			Proteins: Amide I: β-sheet structure
1593			1594	Proteins: Amide II- mainly N-H bending
	1566			Proteins: ring base, phenyl, aromatics
1556			1550	Proteins: Amide II- mainly N-H bending
		1537		Proteins: Amide II- α-helical structure
			1521	Proteins: Amide II- β-sheet structure
	1510			Proteins: Amide II, chain β-sheet structure C=C stretching, aromatics
1481		1483		Lipids: C-H deformation
	1401			Bending mode of CH_3_
1255	1250	1255		Proteins: Amide III- mainly N-H bending, C-N stretching
1222			1220	Phosphates: ν_asym_ PO^2**−**^
	1204	1207		Collagen, C-C stretching, C-H bending, ν_asym_ PO^2**−**^
1191				Vibrational modes of collagen, phosphate band
1103	1105	1110	1107	Carbohydrates, C-O, C-C stretching, ν_sym_ PO^2**−**^
1072				Phosphates: ν_sym_ PO^2**−**^
	1045	1047	1043	Carbohydrates, C-O stretching, glycogen, DNA, RNA
1037				Carbohydrates, C–O stretching
		983		Nucleic acids and proteins, protein phosphorylation, OCH_3_ (polysaccharides-cellulose)
964				Nucleic acids, symmetric PO^4−^ stretching (DNA) and deoxyribose; phosphate skeletal motions
	943		946	Deoxyribose

### Molecular factors

**Figure 5:**
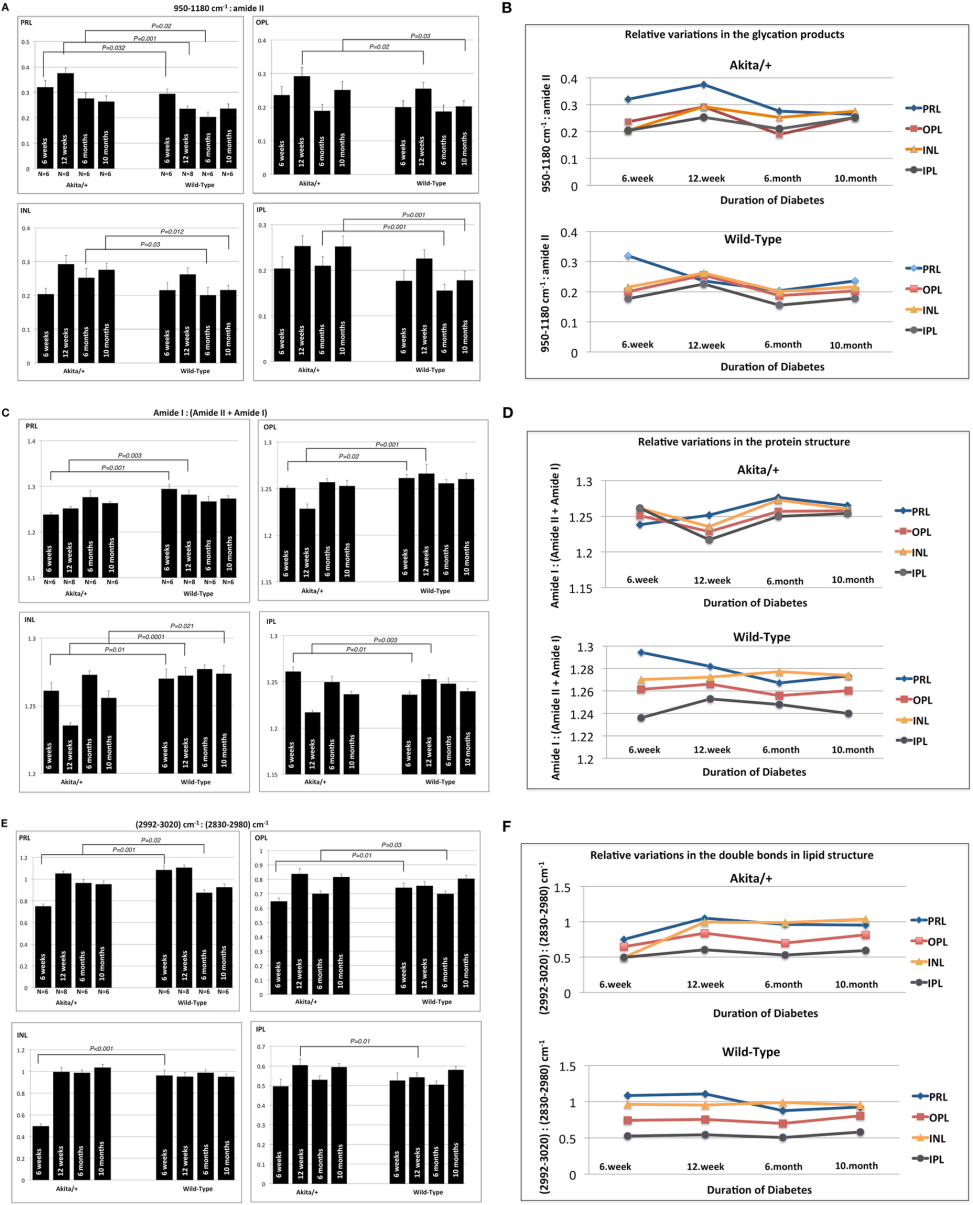
(**A**) Statistical analysis of the ratio measured as the integral of the bands between 950 and 1180 cm^−1^ (C-O, C-C, C-O-C stretches) to the integral of amide II band (1540 cm^−1^) in wild type and Akita/+ mice, as a function of age. Spectral regions 950–1180 cm^−1^and 1500–1750 cm^−1^ were used as baseline. (**B**) Statistical analysis of the ratio measured as the integral of the amide I band (1650 cm^−1^) to the integral of the bands between 1500 and 1700 cm^−1^ (total amide II and I) in wild type and Akita/+ mice, as a function of age. The spectral region between 1500 and 1750 cm^−1^ was used a baseline. (**C**) Statistical analysis of the ratio measured as the area of the olefinic band (2992–3020 cm^−1^) to the C-H region (2830–2980 cm^−1^) in wild type and Akita/+ mice, as a function of age. The spectral region between 2800 and 3050 cm^−1^ was used a baseline. Data are represented as mean ± S.D. Trends highlighting the variations in the degree of glycation (**D**), relative variations in the amide groups of proteins (**E**), and unsaturation level in lipid chains (**F**), as a function of age for the PRL, OPL, INL, and IPL. Student *t*-test was performed for two group comparisons and P < 0.05 were accepted as significantly different. N represents the number of animals. PRL, Photoreceptor retinal layer; OPL, Outer plexiform layer; INL, Inner nuclear layer; IPL, Inner plexiform layer.

As shown (Fig. 5), we computed the spectral metrics (see methods for details) for each retinal layer as a function of diabetes duration to determine the significant relative variations in the macromolecular functional groups between non-diabetic and diabetic mice. The first metric was the ratio of integrated absorbance between 950 and 1180 cm^−1^ (C-O, C-C, C-O-C stretches) to the integrated absorbance between 1510 and 1570 cm^−1^ (amide II). This metric is indicative of the presence of advanced glycation end (AGE) products^36,37^. For all retinal layers and all diabetes durations, this ratio was greater in diabetic groups compared with WT groups (Fig. 5A), although some of the differences were not significantly different. The second metric was the ratio of integrated absorbance between 1600 and 1700 cm^−1^ (amide I-proteins) to the integrated absorbance of total amide I and amide II groups between 1500 and 1700 cm^−1^. This metric was used to show the relative variations in the amide groups of proteins in retinal layers, which typically reflects the changes in the total cellular proteins^38^. Statistical analysis revealed that this ratio at 6 and 12 weeks of age was significantly greater for the non-diabetic PRL, OPL, and INL compared to the corresponding layers in Akita/+ group (Fig. 5C). This ratio was significantly larger in the diabetic IPL at 6 weeks in comparison with WT group. This metric was not significantly different between the layers of WT and Akita/+ mice at 6 months of age.

The last metric was the ratio of integrated absorbance between 2992 and 3020 cm^−1^ (CH = CH; olefinic) to the integrated absorbance between 2830 and 2980 cm^−1^ (mainly C-H stretches of lipids; methyl and methylene groups). This metric was used to show the relative variations in the double bonds in lipid chains within retinal layers^39^. This metric revealed a decrease in all diabetic layers compared to the WT group at the age of 6 weeks (Fig. 5E). At 12 weeks, this metric was greater in the diabetic OPL and IPL; however, there was no significant difference between the WT and diabetic PRL and INL at this age. At the ages of 6, and 10 months, diabetic PRL was the only layer with the greater level of this metric in comparison with WT layers.

The time-lapse trends were shown for all three metrics (Fig. 5B,D,F), calculated for individual diabetic and non-diabetic retinal layers, as a function of diabetes duration. The trends facilitate an understanding of the temporal-dependent variations in molecular parameters of retinal layers.

## Discussion

In the present work, we highlighted the bio-molecular changes of retinal layers, and their potential contribution to pathogenesis of DR, with different duration of diabetes. Precise temporal biomarkers of oxidative stress in the retina with sub-cellular spatial resolution level were obtained and the specific macromolecular functional groups associated with the damage were delineated. Our approach integrates spatially resolved chemical imaging and multivariate analysis to determine the intra-retinal layer bio-molecular changes from the onset to late diabetes (Fig. 3). With short duration of diabetes (6 and 12 weeks), the PRL showed a conspicuous segregation from the rest of the layers (Fig. 3A,B), while the separation between layers looked more clustered with longer duration of diabetes (6 and 10 months) (Fig. 3C,D).

The significance of photoreceptor changes in the pathogenesis of early DR^40,41^ and the contribution of mitochondria in diabetes-induced oxidative damage is well studied^42^. One of the promising features of these results is the capacity to ascertain the macromolecules that promote retinal changes. Important biomarkers at 6 weeks were associated with the stretches and skeletal motions of phosphates, the main spectral markers of double stranded DNA. However, the discriminatory features between retinal layers at 12 weeks were mostly attributable to the amide I and amide II of α-helical and β-sheet structure of proteins (Table 2). Intra-retinal layer biomolecular changes at 6 and 10 months of age were mostly attributed to C-O, C-C, and C-O-C stretches of carbohydrates, DNA, RNA ribose, and glycogen as well as several biomarkers in the secondary structure of proteins. Here, biochemical changes in the protein structures with longer duration of diabetes may resonate with the extracellular and intracellular changes induced by glycation reactions via chemical rearrangement^43^. The glycation products built on amino groups of proteins, and in DNA can lead to molecular cross-links.

We studied the biochemical changes of each layer of retina from mice with different duration of diabetes to better assess the development and progression of DR (Fig. 4). This is a potentially important finding that may be of particular benefit to understand the molecular and biological activity of retinal cells in response to oxidative stress during diabetes, which still remains unclear. When the diabetic retinal layers were studied, the spectra from 12 weeks, 6 months, and 10 months were adjacent clusters in the scores plot; however, the spectra from 6 weeks revealed a clear separation (Fig. 4). This finding implies the uniqueness of damage at 6 weeks within the layers of retina form  Akita/+ mice. Classification between the spectra from IPL at different durations of diabetes (Fig. 4D) revealed major biomarkers attributable to α-helical and β-sheet structures of proteins that suggest the significance of protein changes in this layer with progression of diabetes. These bio-molecular changes may have significant impact on retinal neuronal function early in diabetes.

A limitation of this study was that tracking biochemical changes in the temporal, nasal, inferior, and superior quadrants in each eye was not attainable. It has been shown in clinical data^44^ as well as results of morphological studies on animal models^45^ that the retina does not respond homogeneously to diabetic conditions, but a remarkable regional variation in susceptibility exists. However, spectral acquisition from multiple regions of the retina was adopted in this study (see methods section) to account for the potential differences among diverse regions of the retinal tissue. We believe that quantifying the temporal diabetes-induced changes in the retinal layers in this manner is critical to establish proof of principle. However, it is far beyond the scope of this paper to provide a detailed explanation of the heterogeneities that exist in the retina. A study utilizing eye tracking to detect biochemical alterations in each quadrants of the eye as well as central, paracentral, and peripheral discrimination, would provide highly localized mechanistic information.

The ratio between the areas of bands associated with C-O, C-C, and C-O-C to amide II in proteins provides information on the molecular modifications in the glycation products. It has been shown that the ratio of the absorbance from the sugar moieties to the amide II of proteins indicates the presence of advanced glycation end (AGE) products^36,37^. However, we cannot predict the contribution of the co-located aggregates of lipid esters to the accretion of glycation products. We found that this ratio was significantly greater for the PRL in retinas from 6 weeks, 12 weeks, and 6 months old diabetic mice. However, this ratio was significantly larger for the OPL at 12 weeks and 10 months of age (Fig. 5A). For the INL and IPL, this metric was significantly greater at 6 and 10 months of age, which suggests the impacts of the glycation products within these layers late in diabetes. Time-lapse variations in this ratio, for all diabetic layers, revealed an increase from 6 to 12 weeks, and then a decrease at 6 months followed by another increase at 10 months of age (Fig. 5B). Excessive production of ROS during diabetes leads to an imbalance in these byproducts of metabolism, and increased oxidative stress. One of the major tasks of the cell’s compensatory mechanisms is the repair of the oxidatively damaged or degraded macromolecules. Molecular ROS-scavengers, enzymatic antioxidants, and proteins that help the repair machinery are the known defense mechanisms^46^. Temporal trend of variations in the glycation level at different durations of diabetes in our data may correlate with the antioxidative mechanisms of retinal cells in response to progression of diabetic changes.

The area ratio between amide I and amide I + amide II bands sheds light on structural variations in proteins within retinal layers. In the plexiform layers of diabetic retina, this ratio did not exhibit any significant change from 6 to 10 months of age; however, the nuclear bodies of diabetic retina revealed a decrease from 6 to 10 months (Fig. 5C,D). For diabetic retinal layers, we found that this metric reached a low at 12 weeks and a peak at 6 months; however, the PRL reached a minimum at 6 weeks of age. One possible biological source of these alterations is the AGEs that are found in the extracellular matrix and that potentially modify the matrix proteins impairing matrix-matrix and matrix-cell interactions^47^. Another source of damage are the intracellular proteins, where the formation of AGE has direct impact on their function. Specific binding of the AGE modified proteins to the receptor of AGE has the potential to damage^6,48^. The most interesting result to emerge from the relative changes in glycation products and the variations in the amide groups of proteins, is that these ratios exhibit nearly the same value for all diabetic retinal layers at 10 months, which states the uniformity of the damage to all the layers with longer duration of diabetes.

The modifications in the unsaturated bonds in lipids was evaluated from the area ratio between C = H (double bonds) and methyl and methylene groups. Investigation of this ratio in the diabetic PRL, OPL, INL, and IPL (Fig. 5E,F) suggests the significance of hydrocarbon chain unsaturation with short duration of diabetes (6 and 12 weeks). The decrease in this ratio in diabetic retinal layers, suggests the loss of unsaturated acyl chains of lipids due to an elevated lipid peroxidation in diabetic retina. The oxidation of unsaturated fatty acids is well established and poly-unsaturated fatty acids are more susceptible to peroxidation due to the number of double bonds^49^. Our results are in agreement with several studies with short-duration of diabetes-mediated changes in retinal fatty acid metabolism^50,51^, which revealed a significant decrease in unsaturation of lipids in diabetic retina.

In summary, this is the first report of the nature of diabetes-mediated cellular changes in the biochemistry of distinctive layers of mouse retina. The function of cell types in the retina and their role in the pathogenesis of diabetes has not been yet clearly delineated. Our results show the capacity of spatially-resolved chemical images combined with PCA-LDA in discriminating spectra from diabetic and non-diabetic retinal tissue layers as well as characterizing biomarkers of diabetes in a robust, objective, and reproducible manner. Here, we show that vibrational spectroscopic imaging is ideal not only for early detection, but also for detecting the progression of the neurodegenerative diseases with different durations. However, more studies are required to fully comprehend the susceptibility of the neuro-retina as an early potential target of changes caused by hyperglycemic environment associated with diabetes. The presented method in this study can well find its utility as a complementary approach in vision research to assess the biochemical profile of the retina. Furthermore, this approach can be applied to the assessment of other pathological conditions in the retina, especially concerning mechanistic implications. This work provides a significant rationale for the development of further studies of the role of retinal cells layers in the pathogenesis of DR. We believe that the knowledge of the nature of biochemical changes in specific retinal layers could be critical for the overall development of improved diagnostics in a cross-sectional manner.

## Materials and Methods

### Ethical note

All experiments were carried out in accordance with the Association for Research in Vision and Ophthalmology Statement for the Use of Animals in Ophthalmic and Vision Research and were approved by the Institutional Animal Care and Use Committee of the University of Wisconsin School of Medicine and Public Health. The datasets generated and analysed during the current study are available from the corresponding author on reasonable request.

### Sample preparation

The procedure for sample preparation has been described explicitly elsewhere^23,33^. Briefly, Male C57BL6 mice (Jackson Laboratories) were housed in standard caging with 12 hour light: dark cycle, and food and water provided *ad libitum*. Akita/+ mice spontaneously develop diabetes at 4-weeks of age due to a mutation in their insulin gene. The Akita spontaneous mutation (commonly referred to as MODY; Maturity-Onset Diabetes of the Young) is an autosomal dominant mutation in the insulin II gene (Ins2). Ins2 ^Akita/+^-C57BL/6 diabetic mice develop retinal vascular pathology characteristic of the early stages of DR. Once sacrificed, eyes were enucleated from male WT and Akita/+ mice at the ages of 6 weeks (N = 6), 12 weeks (N = 8), 6 months (N = 6), and 10 months (N = 6). N refers to number of animals. Eyes were rapidly frozen in isopentane cooled to almost freezing in liquid nitrogen and later stored at −80 °C freezer for FT-IR chemical imaging.

Eyes were embedded in optimal cutting medium (O.C.T) compound and carefully cut into 5–8 μm thin coronal sections using cryomicrotome. Coronal sectioning of the eye started from the optic nerve (from the back of the eye) and the retinas were extracted using a thin paintbrush. The temperature inside the cryomicrotome was maintained between −15 °C and −20 °C and the blade was pre-chilled for at least half an hour prior to sectioning. At least five coronal retinal sections were extracted from each eye and mounted on barium fluoride window. The retinal tissue sections were randomly selected to generate data from several regions of the retina. Six FT-IR wide-field measurements were performed on each tissue section; then the spectra from all tissue sections that were mounted on the window were combined for the analysis. This methodology was used to record data from various regions of the retinal tissue, providing a better assessment of the biochemical alterations in the tissue. All tissue sections were stored in darkness and kept frozen at −80 °C until FT-IR measurements. Before the experiment, the sample was removed from the freezer and thawed. The experiment was performed in dark and low humidity conditions. Schematic of the methodology is shown in Fig. 1.

### Fourier transform infrared (FT-IR) chemical imaging

The FT-IR chemical images were recorded with the use of a Bruker vertex 70 IR spectrometer coupled with a Bruker Hyperion 3000 IR microscope. Hyperspectral images (x, y, Abs (λ)) were acquired by means of the focal plane array (FPA) detector, which is a multielement detector and it is coupled with the interferometer. The measurements were performed using a 36 × (N.A = 0.5) Cassegrain microscope objective and a 15 × condenser aperture. This experimental geometry allows us for high magnification imaging capabilities with a 1.1 × 1.1 μm^2^ pixel size and a 70 × 70 μm^2^ field of view (FOV). We used 2048 scans co-added at a spectral resolution of 4 cm^−1^ with a zero filling factor 2 for background and for sample acquisitions, respectively. To cover a larger area of the tissue, adjacent tiles were measured by mapping across the section of the retina. The FPA size for the measurements was set to 64 × 64 pixels; therefore, 4096 individual spectra in the mid-IR wavelength range 3800–900 cm^−1^ (every pixel contains an IR spectrum) were collected per single tile measurement.

### Selection of the spectra from retinal layers for multivariate data analysis

Analysis of the chemical images from each retinal tissue section was performed using the software package IRidys (a software written on the basis of IgorPro). Pixelated spectra representing areas of 1.1 × 1.1 μm^2^ in the tissue were measured and analyzed. Chemical images derived from distinctive spectral features delineate the retinal layers, since the overall chemistry of each layer is different, and has been identified through prior experimental results^18^. Spectra from pixels within the delineated retinal layers were chosen for the multivariate analysis. Spectra from the pixels of interest were selected via an automated procedure (built-in IGOR algorithm) by defining the X and Y positions of the pixels within each retinal layer, and generated a subset from the cube of data. Selected dataset from each layer were loaded for a stringent signal to noise ratio test and pre-processing to remove spurious data and prepare dataset for spectral classification (as detailed in the pre-processing section).

### Spectral pre-processing

Resultant spectra from tissues revealed Mie scattering distortions. This distortion originates from the scattering of IR light from particles with diameters in the range of 2–20 μm^52,53^. Although the dispersion artefact appears as a derivative shape in the spectral region close to the protein band (Amide I band), this phenomenon could influence spectrum in several spectral regions, and leads to misinterpretation of the chemistry and pathology. In this study, nearly 82,000 individual pixel spectra from each animal were generated and were subjected to the RMies-EMSC scattering correction algorithm to alleviate the contribution of the scattering effect. High-throughput computations of hundreds of thousands of spectra were supported by Graphics Processing Units (GPUs) for fast hyperspectral processing^54^. Once corrected, the spectra were quality tested and preprocessed as follows. The spectra were truncated to 900–1800 cm^−1^ range (finger print region) that resulted in 485 data points. Pixel binning (2 × 2; average of 4 pixels) was applied, which was necessary to increase the signal to noise ratio, and S/N in every spectrum was then systematically evaluated (in-house algorithm in Matlab_R2016a). The CO_2_ peak at 2350 cm^−1^ was flattened between 2500 and 2200 cm^−1^. The baseline was corrected by fitting a linear regression line to spectral points in the 2692–1920 cm^−1^ region, and subtracting that line from the spectrum. Then, the signal to noise ratio of every spectrum was systematically assessed by defining the noise content as the standard deviation in the 2000–1900 cm^−1^ spectral region, and the signal as the maximum of the signal in the 1700–1600 cm^−1^ region. All spectra were vector-normalized prior to computational analysis.

### Multivariate and statistical analysis

The multivariate analysis was performed using in-house written algorithm for Python (2.6 version). Principal component analysis (PCA) was used for the dimensionality reduction and then the optimum number of PCs that showed the greatest variance between the classes were retained (determined through iterative process of validation) and loaded into linear discriminant analysis (LDA). Assigning data to multiple classes using PCA enables an algorithm to determine the sources of within-class and between-class variances in the dataset. The outcome of PCA-LDA is an improved classification between spectra by maximizing between-class variances (mostly attributed to disease) and minimizing within-class variances (mostly attributed to tissue heterogeneity). The algorithm was tested using cross-validation for accurate classification. To test the classifier, we randomly split the spectra from all pre-processed data on 80%−20% for training and further test tests. After testing the classifier using the attributed training set, we can estimate the misclassification error. The details of PCA-LDA construction and the interpretation of results are described elsewhere^55^.

The statistical data were presented as the means ± Standard Deviations. The significance of the molecular factors in samples was calculated using Student *t-*test (two tailed) in SAS statistical software (Version 9.1.3, SAS Institute Inc., Cary, NC, USA) and P < 0.05 was considered significant.

### Spectroscopic parameters

The ratio of the integrated area of the spectral region attributed to C-O, C-C, and C-O-C stretches (1180–950 cm^−1^) to the integrated area of the amide II band (1510–1580 cm^−1^) of proteins was calculated. The former spectral region entails significant vibrational modes of sugar moieties, phosphates, and glycation products. This ratio indicates the relative changes in the glycation products in the retinal layers^36,37^. The relative alteration in the structure of amide groups of proteins was calculated as a ratio of the integrated area of amide I band (1600–1700 cm^−1^) normalized to the total amide I and amide II profile (1500–1700 cm^−1^). The changes in this ratio provide information about the modifications in the secondary structure of proteins^38^. The degree of unsaturation in the lipid chains was assessed by calculating the ratio of the area of the olefinic band (2992–3020 cm^−1^) to the C-H region (2830–2980 cm^−1^), mainly dominated by methyl and methylene groups. This ratio indicates the relative content of unsaturated lipids and double bonds in the lipid structure of the tissue^39^. The spectral regions 1500–1750 cm^−1^ (for amide I and II bands), 950–1180 cm^−1^ (for C-O, C-C, and C-O-C bands), and 2800–3050 cm^−1^ (for olefinic and C-H bands) were used as a baseline region.
